# Transplacental Transmission of Bluetongue Virus Serotype 1 and Serotype 8 in Sheep: Virological and Pathological Findings

**DOI:** 10.1371/journal.pone.0081429

**Published:** 2013-12-16

**Authors:** Mirjam T. W. van der Sluijs, Dianne P. H. Schroer-Joosten, Aicha Fid-Fourkour, Mieke P. Vrijenhoek, Isolde Debyser, Véronique Moulin, Rob J. M. Moormann, Abraham J. de Smit

**Affiliations:** 1 Research and Development, MSD Animal Health, Boxmeer, The Netherlands; 2 Animal Services and Pathology, MSD Animal Health, Boxmeer, The Netherlands; 3 CoVeToP, Consultancy in Veterinary and Toxicological Pathology, Enghien, Belgium; 4 Central Veterinary Institute, Animal Sciences Group, Wageningen University and Research centre, Lelystad, The Netherlands; 5 Department of Infectious Diseases and Immunology, Virology Division, Faculty of Veterinary Medicine, Utrecht University, Utrecht, The Netherlands; Wageningen University and Research Centre, The Netherlands

## Abstract

The Bluetongue virus serotype 8 (BTV-8) strain, which emerged in Europe in 2006, had an unusually high ability to cause foetal infection in pregnant ruminants. Other serotypes of BTV had already been present in Europe for more than a decade, but transplacental transmission of these strains had never been demonstrated. To determine whether transplacental transmission is a unique feature of BTV-8 we compared the incidence and pathological consequences of transplacental transmission of BTV-8 to that of BTV-1. Nine pregnant ewes were infected with either BTV-8 or BTV-1. The BTV strains used for the infection were field strains isolated on embryonated chicken eggs and passaged twice on mammalian cells. Blood samples were taken to monitor the viraemia in the ewes. Four weeks after the infection, the foetuses were examined for pathological changes and for the presence of BTV. BTV-8 could be demonstrated in 12 foetuses (43%) from 5 ewes (56%). %). BTV-1 was detected in 14 foetuses (82%) from 6 ewes (67%). Pathological changes were mainly found in the central nervous system. In the BTV-8 group, lympho-histiocytic infiltrates, gliosis and slight vacuolation of the neuropil were found. BTV-1infection induced a severe necrotizing encephalopathy and severe meningitis, with macroscopic hydranencephaly or porencephaly in 8 foetuses. In our experimental setting, using low passaged virus strains, BTV-1 was able to induce transplacental transmission to a higher incidence compared to BTV-8, causing more severe pathology.

## Introduction

Bluetongue virus (BTV), an orbivirus (*Reoviridae*), is the causative agent of bluetongue, a severe haemorrhagic disease of ruminants (primarily sheep) characterized by fever, oedema, mucosal erosions and coronitis [1], [2]. BTV has a double-stranded RNA genome, comprised of 10 segments that encode structural proteins VP1 to VP7 and non-structural proteins NS1 to NS4 [3], [4]. BTV is a heterogeneous virus species: 24 serotypes (determined by the outer shell protein VP2 and, to a lesser extent, VP5) have been described to date; recent isolates from Switzerland and Kuwait have been proposed as 25^th^and 26^th^ serotype [5], [6]. Even within one serotype, large genetic and phenotypic variations can be observed related to geographical origin : BTV topotypes [7], [8]. The ability of BTV strains to reassort genome segments adds to the variability between virus strains [9]–[11] and may even result in BTV strains with enhanced virulence [12] or increased abilities to adapt to new ecological zones [10].

The main BTV transmission route is through biting midges of the *Culicoides* species. Other routes, like transplacental transmission, are probably of less epidemiological importance. However, the impact of transplacental transmission on agro-economic parameters such as fertility and reproductive performance can be substantial [13], [14]. Transplacental transmission of BTV had already been described in 1955 [15], when vaccination of pregnant dams with a live embryonated-chicken-egg-passage attenuated BTV vaccine led to the birth of lambs with serious nervous system defects. This has been reproduced in both sheep and cattle in an experimental setting, using various BTV serotypes [16]–[20]. In most of these experiments, BTV strains were used, which had been given a high number of *in vitro* -or *in ovo*- passages, In contrast, low-passaged strains did not cross the placenta [21]. This led to the assumption that transplacental transmission of BTV was exclusively associated with cell culture- or egg-adapted BTV strains [22].

During the BTV-8 outbreak in Europe (2006–2009), transplacental transmission of BTV-8 was reported in several countries; with incidence ratios up to 33% [23]. Prior to this BTV-8 outbreak, BTV-1, BTV-2, BTV-4, BTV-9, and BTV-16 had already been present in southern Europe [9]. Except for the live BTV-2 and BTV-9 vaccine strains, for which a 2.4% incidence of vaccine virus positive foetuses has been demonstrated [24], transplacental transmission of these other BTV strains has not been described in Europe.

The aim of our study was to compare the incidence and pathological consequences of transplacental transmission of BTV-8 to that of another European BTV strain: BTV-1 [25].

We demonstrated that a European isolate of BTV-1, which has had one passage on embryonated chicken eggs (ECE) and two passages on mammalian cells, is also capable of establishing an infection in ovine foetuses. The incidence of transplacental transmission of BTV-1 was at least as high as that of BTV-8. Moreover, BTV-1 induced severe central nervous system defects such as hydranencephaly, whereas these lesions were not observed in the BTV-8 infected foetuses.

## Materials and Methods

### Ethics statement

The experiment was performed in accordance with European Community guidelines and national laws on animal experiments. The design of the experiment was approved by the MSD Animal and Human Health Committee on the Ethics of Animal Experiments (‘Dierexperimentencommissie’, which is required by national legislation to include both MSD AH employees and independent members) prior to the start (Permit Number: BTV 11.054). All efforts were made to minimize animal discomfort.

### Animals

Twenty Swifter ewes were synchronized using medroxyprogesterone acetate intravaginal sponges (Veramix, Pfizer) in January 2012. On the day of removal of the sponges, the ewes received 750 IU pregnant mare serum gonadotrophin (Folligonan, MSDAH) and three rams were added to the flock. The ewes were checked for pregnancy at regular intervals. Nine weeks after mating, the ewes were transferred from the conventional stables to two rooms of the MSD Animal Health (MSDAH) isolation facilities. Each room contained nine BTV inoculated ewes and one control ewe. The animal room was treated with cyfluthrin (Solfac, Bayer) shortly before housing of the animals.

Blood samples, taken from the ewes just before BTV-infection, were free from Bluetongue virus, ruminant pestiviruses and antibodies against those viruses. Blood samples taken prior to the synchronization had high Schmallenberg virus (SBV) neutralizing antibody titres, indicating that an SBV infection had taken place prior to the synchronization of the ewes.

### Design of the experiment

Twenty pregnant ewes were enrolled in the experiment. At approximately 70–75 days gestation, nine randomly chosen ewes were inoculated with 4.0 log_10_ TCID_50_/10 ml BTV-8, nine ewes received 4.0 log_10_ TCID_50_/10 ml BTV-1 and two remaining ewes served as untreated controls. The inocula were administered subcutaneously in the axillary fold.

The ewes were observed once daily for clinical signs of BTV infection (including body temperatures) and for signs of abortion. Blood samples for antibody and virus detection were taken prior to inoculation and every 2–3 days until the end of the experiment.

Twenty-nine days after inoculation, all ewes were euthanized. The ewes were inspected for macroscopic lesions and samples of the spleens were taken for virus isolation. Samples were taken from the foetuses' umbilical cord blood, placentomes (one for each foetus), spleen, liver, kidney, thymus, tongue, cerebrum and cerebellum. Each of the tissue samples was taken in duplicate: one sample was fixated in 4% buffered formaldehyde, embedded in paraffin and stained with haematoxylin and eosin for microscopic pathology; the other sample was stored at −70°C for virus isolation. To avoid cross-contamination, the placebo-infected ewes were autopsied first and the equipment was disinfected between the sampling of the foetuses.

### Inocula

The Bluetongue virus serotype 8 strain was isolated from a clinically affected ewe during the 2006 epidemic in the Netherlands. The BTV-1 strain was isolated from a clinically affected sheep in Spain (kindly supplied by M. Domingo-Alvarez of the Centre de Recerca en Sanitat Animal (CReSA), Barcelona, Spain).

Both viruses were isolated on ECE and subsequently passaged once on baby hamster kidney cells (BHK21) and once on Vero cells, to obtain virus stocks OvBTV-8 Ver1291007 07K07 and BTV-1 MSl22092009. The virus stocks were free from bacterial and viral contaminants. Both virus stocks were diluted in cell culture medium to a titre of 3.0 log_10_ TCID_50_/ml briefly before the inoculation of the pregnant ewes.

### Virus isolation (VI) from blood and tissue samples

EDTA blood samples were washed and sonicated as described earlier [26]. Approximately 0.5 cm^3^ of tissue sample was homogenized in 2 ml culture medium containing antibiotics and antifungals. A sample of 1 ml of sonicated blood or tissue homogenate was incubated for 1–1.5 h on a 25 cm^2^ sub-confluent (70–90%) monolayer of Vero cells. Subsequently, the sample was removed and the monolayers were washed once with PBS to prevent toxicity to the monolayer. The monolayers were incubated at 37°C and 5% CO_2_ for 7 days and regularly examined for the presence of cytopathogenic effect (CPE). If no CPE was visible, the monolayers were propagated using trypsin and again incubated for 7 days. This step was repeated until three cell culture passages had been performed.

To determine the BTV serotype of positive VI test samples, serotype specific PCR tests were performed on the supernatant as described below.

### Detection of viral RNA in blood and cell culture supernatant

Viral RNA was extracted from duplicates of 200 µl of sample using the MagNA Pure 96 System extraction robot (Roche Diagnostics) and the MagNA Pure 96 DNA and Viral NA Small Volume Kit. RNA was eluted in 50 µl and stored at −70°C until use.

#### panBTV RT-qPCR

The real-time reverse transcriptase polymerase chain reaction (RT-qPCR) designed to detect all serotypes of BTV was used ([27]with minor modifications). From each of the RNA extracts, 6 µl was tested using the SuperScript III Platinum One-Step Quantitative RT-PCR system (Invitrogen). Individual cycle threshold (Ct) values were determined at the point at which the level of fluorescence passed a threshold; the PCR Base Line Subtracted Curve Fit method (CFX96 software, BioRad) was used to analyse the data. Ct values of duplicates were averaged; 46.00 was used for calculations when no Ct value was obtained. Average Ct values were categorized as negative (Ct≥35.00), doubtfully positive (Ct between 30.00 and 35.00) or positive (Ct<30.00), as described before [28].

#### Serotype specific RT-qPCRs

For the RT-qPCR to specifically detect either BTV serotype 1 or 8, the LSI VetMax European BTV typing (1-2-4-6-8-9-11-16) Real Time PCR kit (Cat. No. BTVEUG, LSI/Life Technologies) was used in accordance with the manufacturer's instructions.

### BTV specific antibody tests

The VMRD® competitive inhibition antibody ELISA was used in accordance with the manufacturer's instructions to test for the presence of BTV-specific antibodies.

## Results

### Ewes

At the time of the infection, all ewes were free from BTV (Table 1). All BTV infected ewes were BTV RNA positive at 5 days post infection (dpi), except for ewe 78654 (BTV-8 group), which was positive at 7 dpi. The BTV infected ewes remained viraemic until the time point of necropsy, 29 dpi. The non-infected control ewe (Number 78600) that was housed in the same pen as the BTV-8 infected ewes remained BTV RNA negative throughout the experiment. In blood samples from control ewe 78626 (housed together with the BTV-1 infected ewes) BTV RNA could be demonstrated at 21 and 26 dpi. Presence of BTV at the time of necropsy was confirmed by isolation of BTV virus from a sample of the spleen of control ewe 78626; serotype specific RT-qPCRs confirmed the presence of BTV-1.

**Table 1:** Presence of Bluetongue virus RNA and BTV antibodies in blood samples of ewes on different days post inoculation.

		RT-qPCR at day post infection^a^								BTV antibodies at day post infection^b^	
Group	Ewe number	0^c^	5	7	11	14	21	26	29^d^	0^c^	29^d^
BTV8	5572	46.00	33.82	**27.13**	**28.16**	**28.90**	**29.12**	**30.01**	**28.74**	−	+
	78612	46.00	**24.76**	**19.76**	**20.45**	**23.61**	**23.48**	**24.36**	**23.54**	−	+
	78613	46.00	31.54	**26.19**	**21.69**	**22.39**	n.s.^e^	n.s.	n.s.	−	ns
	78618	46.00	**22.02**	**21.82**	**23.85**	**25.37**	**25.66**	**26.42**	**25.44**	−	+
	78648	46.00	**23.33**	**21.44**	**21.97**	**23.91**	**24.25**	**25.86**	**23.77**	−	+
	78653	46.00	31.69	**27.30**	**21.64**	**21.79**	**23.81**	**25.41**	**25.15**	−	+
	78654	46.00	35.41	**29.45**	**22.26**	**24.98**	**26.00**	**26.43**	**25.36**	−	+
	78676	46.00	**22.98**	**20.71**	**21.35**	**21.77**	**23.27**	**24.79**	**25.06**	−	+
	78678	46.00	**29.69**	**24.62**	**21.14**	**21.94**	**24.39**	**24.96**	**23.94**	−	+
BTV1	5537	46.00	**24.43**	**19.93**	**21.43**	**21.46**	**23.58**	**23.08**	**22.29**	−	+
	5566	46.00	**24.24**	**19.50**	**19.86**	**20.77**	n.s.	n.s.	n.s.	−	ns
	5585	46.00	**25.23**	**20.76**	**23.16**	**23.21**	**24.24**	**23.59**	**23.59**	−	+
	78630	46.00	**22.85**	**21.86**	**21.91**	**25.40**	**26.57**	**25.60**	**25.37**	−	+
	78631	46.00	**24.12**	**20.23**	**19.79**	**21.53**	**25.05**	**24.32**	**23.66**	−	+
	78634	46.00	**26.23**	**19.77**	**19.74**	**23.82**	**24.67**	**23.78**	**23.12**	−	+
	78649	46.00	**23.74**	**19.95**	**19.59**	**23.69**	**24.67**	**23.73**	**23.51**	−	+
	78655	46.00	**27.73**	**22.58**	**19.96**	**20.46**	**23.02**	**23.66**	**23.68**	−	+
	78656	46.00	**22.85**	**21.46**	**20.47**	**22.12**	**26.17**	**26.41**	**23.47**	−	+
Control BTV-8	78600	46.00	46.00	46.00	46.00	46.00	46.00	46.00	46.00	−	−
Control BTV-1	78626	46.00	43.10	46.00	46.00	46.00	**25.16**	**21.70**	n.s.^f^	−	+

= positive (in bold), 30≤Ct<35 = doubtful, Ct≥35 = negative.^a^ RT-qPCR result: Ct<30

+ antibodies detected; −: no antibodies detected.^b^ BTV antibodies:

^c^ before inoculation,

^d^ at necropsy.

^e^ n.s.: no sample.

^f^ no EDTA sample was available from ewe 78626 at the time of necropsy.

However, BTV-1 could be isolated from the spleen sample of this ewe.

All ewes were BTV antibody negative at the time of infection. Four weeks later, at the time of necropsy, all 18 BTV infected ewes had formed BTV antibodies. The control ewe in the BTV-8 group remained negative until the end of the experiment. However, the control ewe in the BTV-1 group developed antibodies against BTV (Table 1).

The BTV-8 infection induced mostly mild clinical signs like fever, rhinitis, conjunctivitis, hyperaemia, and facial oedema. Ewe 78613showed anorexia, dyspnoea, and severe lameness at 18 dpi and it was euthanized (sodium pentobarbital (Euthazol 40%, AST Pharma)) for animal welfare reasons.

The BTV-1 infection resulted in more severe clinical signs: 4/9 ewes showed severe dyspnoea, fever, anorexia, foaming at the mouth, oral lesions, lameness, and facial oedema. Ewe 5566 was euthanized at 15 dpi because of anorexia and severe respiratory distress: necropsy showed that the ewe suffered from a multifocal pneumonia.

### Foetuses

#### Virology

The nine ewes in the BTV-8 group had 28 foetuses; 12 foetuses (43%) from 5 ewes (56%) were positive for BTV in the panBTV RT-qPCR on samples of the umbilical cord blood (Table 2). The positive results of the RT-qPCR were confirmed by isolation of BTV from the blood and spleen samples. In 11 of 12 positive foetuses, BTV could also be isolated from the cerebrum, the cerebellum, or both. Serotype specific RT-qPCRs confirmed the presence of BTV-8 and the absence of BTV-1 in the BTV-8 group. From two of the s foetuses 78613.2 and 78613.3, no umbilical cord blood samples could be taken, since the blood had already clotted. However, spleen samples were negative in the VI test so these foetuses were considered uninfected.

**Table 2:** Presence of Bluetongue infectious virus or RNA in foetal tissues: BTV 8 infected group+commingled control.

		Foetal tissues				
		Umbilical cord blood^a^		Spleen	Cerebrum	Cerebellum
Group	Foetus number	VI	RT-qPCR	VI	VI	VI
Uninfected controls	78600.1	−	46.00	−	−	−
BTV8 infected	5572.1	−	46.00	−		
	5572.2	−	46.00	−		
	5572.3	−	40.75	−		
	5572.4	−	46.00	−		
	78612.1	+	**16.20**	+	+	+
	78612.2	−	46.00	−		
	78613.1	−	42.52	−		
	78613.2	n.s.	n.s.	−		
	78613.3	n.s.	n.s.	−		
	78618.1	−	46.00	−		
	78648.1	−	**19.94**	+	−	+
	78648.2	+	**21.57**	+	+	+
	78648.3	+	**20.54**	+	+	+
	78648.4	+	**23.88**	+	+	+
	78653.1	−	46.00	−		
	78653.2	−	46.00	−		
	78653.3	−	42.03	−		
	78653.4	−	41.72	−		
	78653.5	−	46.00	−		
	78654.1	+	**16.80**	+	+	+
	78654.2	+	**17.08**	+	+	+
	78654.3	+	**27.48**	+	−	−
	78654.4	+	**22.76**	+	−	+
	78676.1	+	**18.06**	+	+	+
	78676.2	+	**16.27**	+	+	+
	78676.3	+	**18.30**	+	+	+
	78678.1	−	46.00	−		
	78678.2	−	46.00	−		
Total no. of pos samples/no. of samples tested		11/24	12/26	12/28	9/12	11/12

= positive (in bold), 30≤Ct<35 = doubtful, Ct≥35 = negative.^a^ RT-qPCR result: Ct<30

Virus isolation (VI): + cpe detected; −: no cpe detected. ns: no sample.

The BTV-8 control ewe 78600 had only one foetus; this foetus was BTV negative (Table 2).

The nine ewes in the BTV-1 group had 17 foetuses. Six BTV-1 infected ewes (67%) transmitted the virus to their foetuses, resulting in 14 BTV RT-qPCR positive foetuses (82%). The positive panBTV RT-qPCR results were confirmed by virus isolations from the spleen, cerebrum or cerebellum (Table 3). Serotype specific RT-qPCRs confirmed the presence of BTV-1 and not BTV-8 in the foetuses.

**Table 3:** Presence of Bluetongue infectious virus or RNA in foetal tissues: BTV-1 infected group+commingled control.

		Foetal tissues				
		Umbilical cord blood^a^		Spleen	Cerebrum	Cerebellum
Group	Foetus number	VI	RT-qPCR	VI	VI	VI
Uninfected control	78626.1	−	46.00	−	−	−
	78626.2	−	46.00	−	−	−
	78626.3	−	46.00	−	−	−
	78626.4	−	46.00	−	−	−
	78626.5	−	46.00	−	−	−
BTV1 infected	5537.1	−	46.00	−		
	5566.1	−	35.67	−		
	5585.1	−	46.00	−		
	78630.1	+	**19.03**	+	+	−
	78630.2	+	**20.68**	+	+	+
	78631.1	+	**19.06**	+	+	+
	78631.2	+	**21.49**	+	+	+
	78631.3	+	**24.58**	−	+	+
	78634.1	+	**22.45**	+	+	+
	78634.2	+	**27.13**	−	+	−
	78649.1	+	**22.45**	−	+	+
	78649.2	+	**22.54**	+	+	+
	78655.1	−	**20.27**	+	+	+
	78655.2	+	**20.15**	−	+	+
	78655.3	+	**25.81**	−	+	+
	78656.1	+	**21.95**	+	+	−
	78656.2	+	**18.93**	+	+	+
Total no. of pos samples/no. of samples tested		13/17	14/17	9/17	14/14	11/14

= positive (in bold), 30≤Ct<35 = doubtful, Ct≥35 = negative.^a^ RT-qPCR result: Ct<30

Virus isolation (VI): + cpe detected; −: no cpe detected. ns: no sample.

Although the BTV-1 control ewe (No. 78626) became BTV RNA positive between 14 and 21 dpi, all tests on the tissues of the foetuses were negative, indicating that the virus was not transmitted to the foetuses (Table 3).

#### Pathology

**Figure 1:**
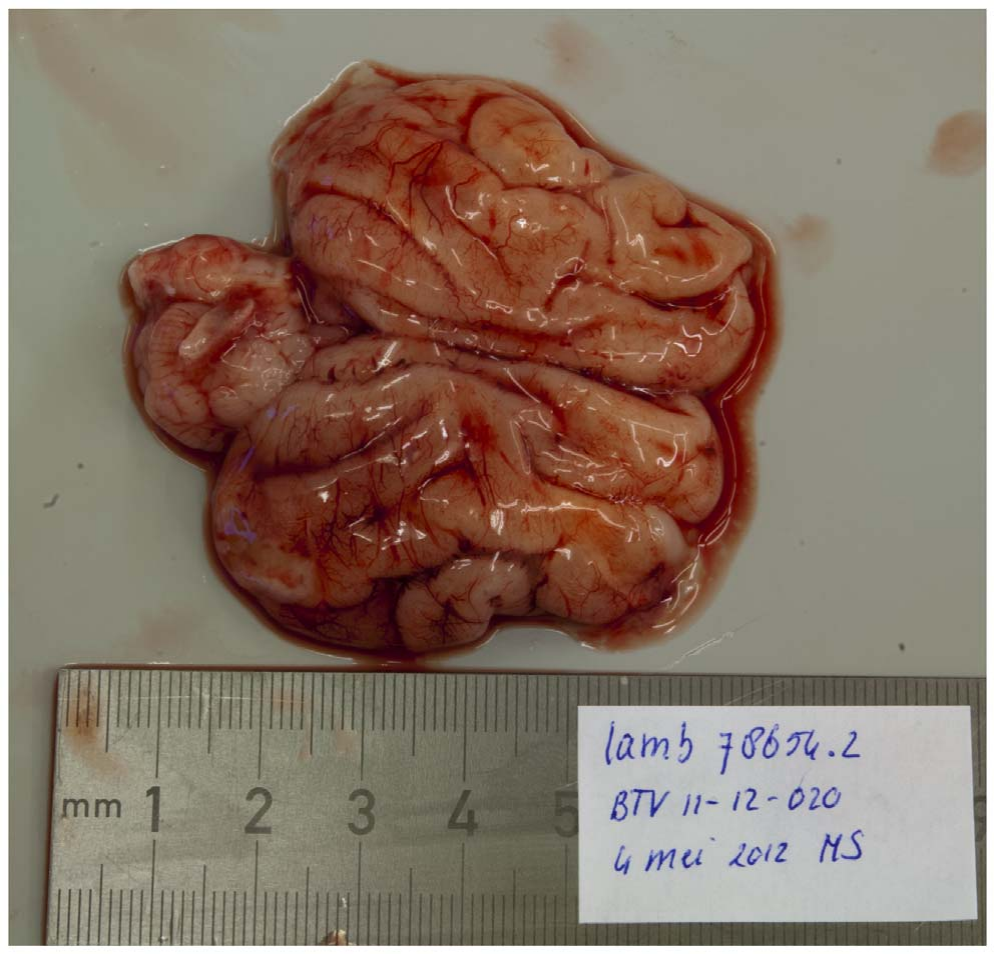
Normal foetal brain with normally developed cerebral gyri. Foetus 78654.2 (BTV-8 group, VI/PCR positive).

**Figure 2:**
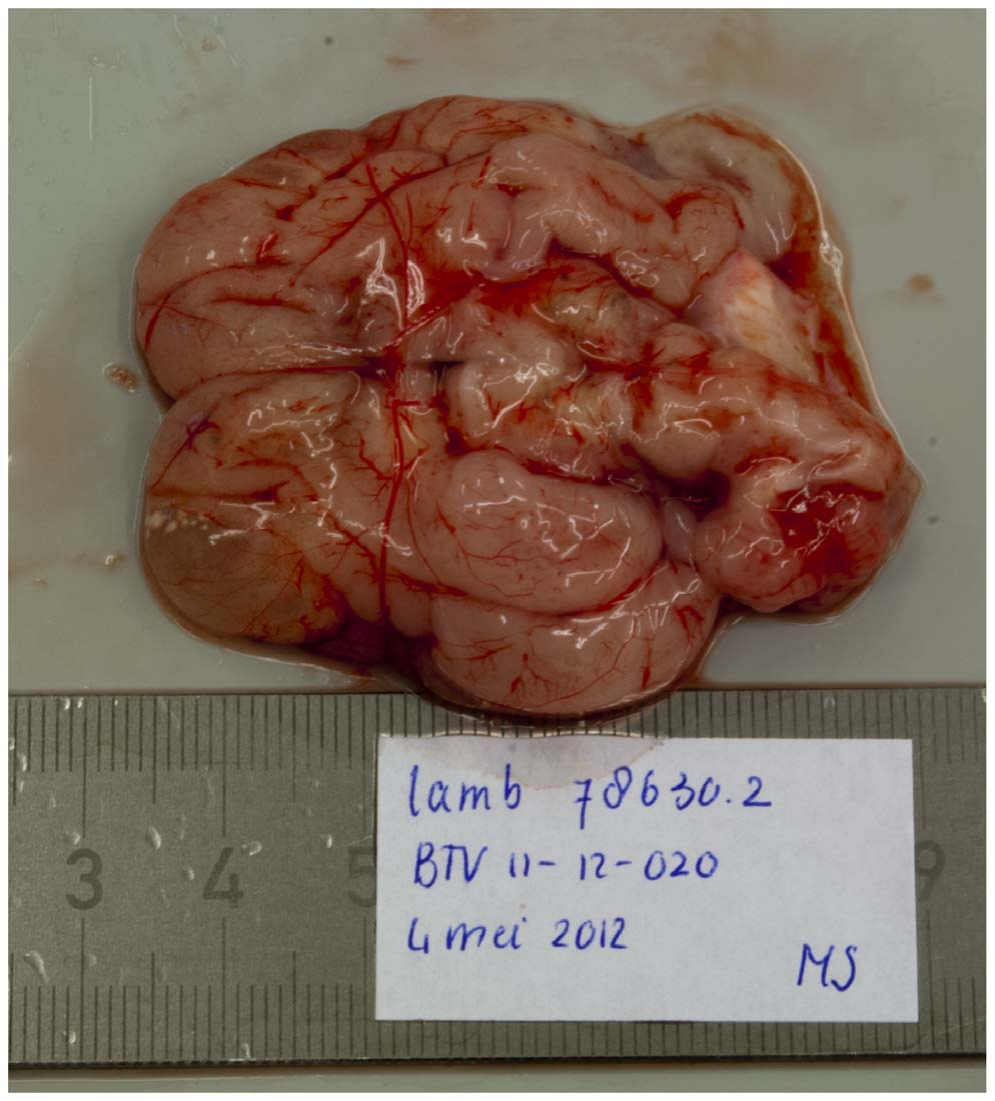
Localized cystic defect in the cerebral hemisphere (right frontal lobe). Foetus 78630.2 (BTV-1 group, VI/PCR positive).

**Figure 3:**
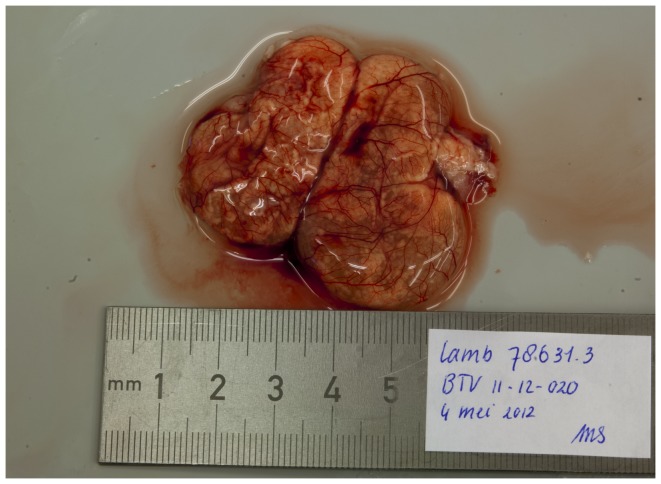
Hydranencephaly. The cerebrum is transformed to fluid filled sacs with only focal presence of cerebral tissue at the periphery. Foetus 78631.3 (BTV-1 group, VI/PCR positive).

The most prominent macroscopic pathological findings in the foetuses were central nervous system lesions. In a normally developed foetal brain, gyral convolutions can already be seen at this stage (Figure 1). In seven BTV-1 infected foetuses hydranencephaly was observed: the cerebral hemispheres were transformed to membranous sacs filled with a clear, reddish fluid. Occasionally, small islands of remaining cerebral tissue could be seen in the wall of these membranous sacs (Figure 2). In foetus 78630.2, porencephaly was observed: localized cystic defects in the cerebral hemispheres (Figure 3).

These severe macroscopic central nervous system deformations were only seen in foetuses from the BTV-1 group.

**Figure 4:**
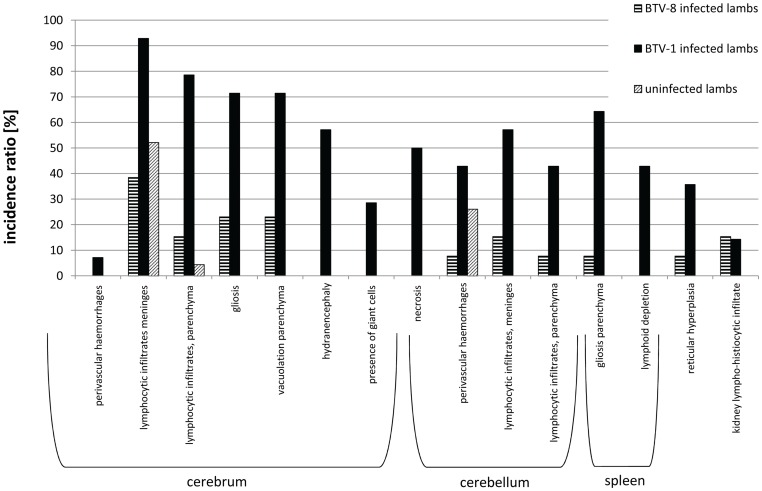
Incidence ratio of microscopic findings. Incidence of lesion per number of foetuses for the BTV-8 positive foetuses (n = 14), BTV-1 positive foetuses (n = 14) and BTV negative foetuses (n = 23).

To evaluate the incidence of microscopic lesions, the incidence ratios of microscopic lesions in the BTV-8 positive foetuses, the BTV-1 positive foetuses, and the negative foetuses were compared (Figure 4). Inflammation of the central nervous system was the most prominent microscopic finding in both the BTV-8 and the BTV-1 infected foetuses.

In the BTV-8 group, the central nervous system lesions consisted mainly of lympho-histiocytic infiltrates and gliosis. Slight vacuolation of the neuropil was seen in foetuses 76854.1, 2 and 3. In the spleen, slight reticular hyperplasia was seen in 1 foetus and lympho-histiocytic infiltrates in the kidney were seen in 2 foetuses.

**Figure 5:**
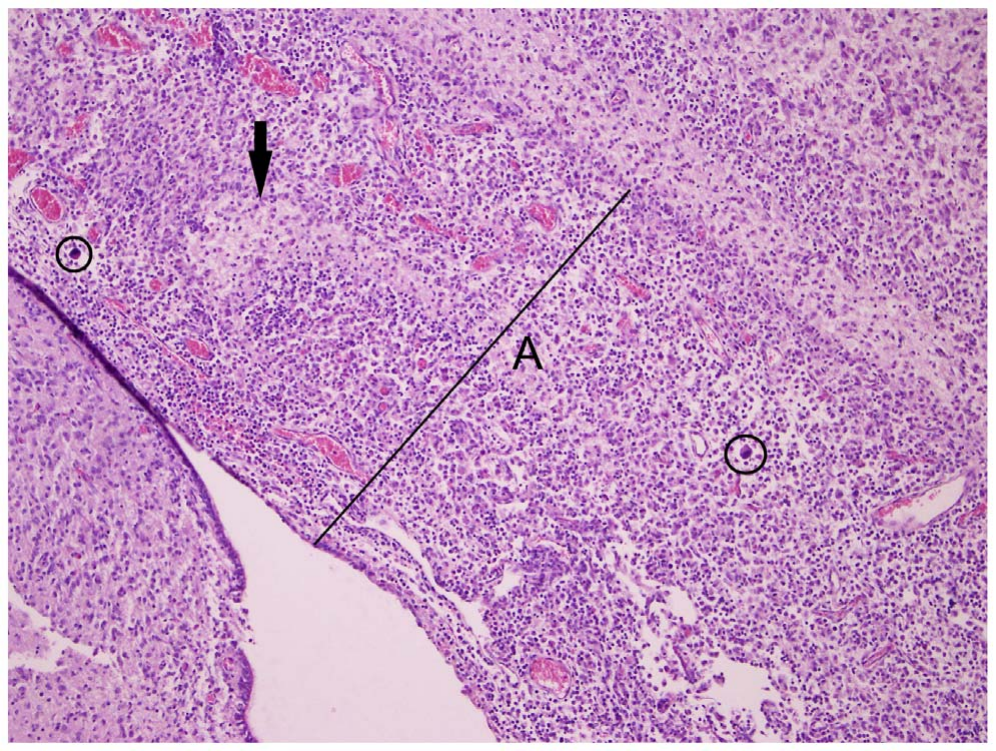
Meningitis. Increase of meninges thickness (A) due to mononuclear infiltrate, necrosis (arrow) and presence of giant cells(circle). Cerebrum (10×10) of foetus 78649.1 (BTV-1 group, VI/PCR positive).

**Figure 6:**
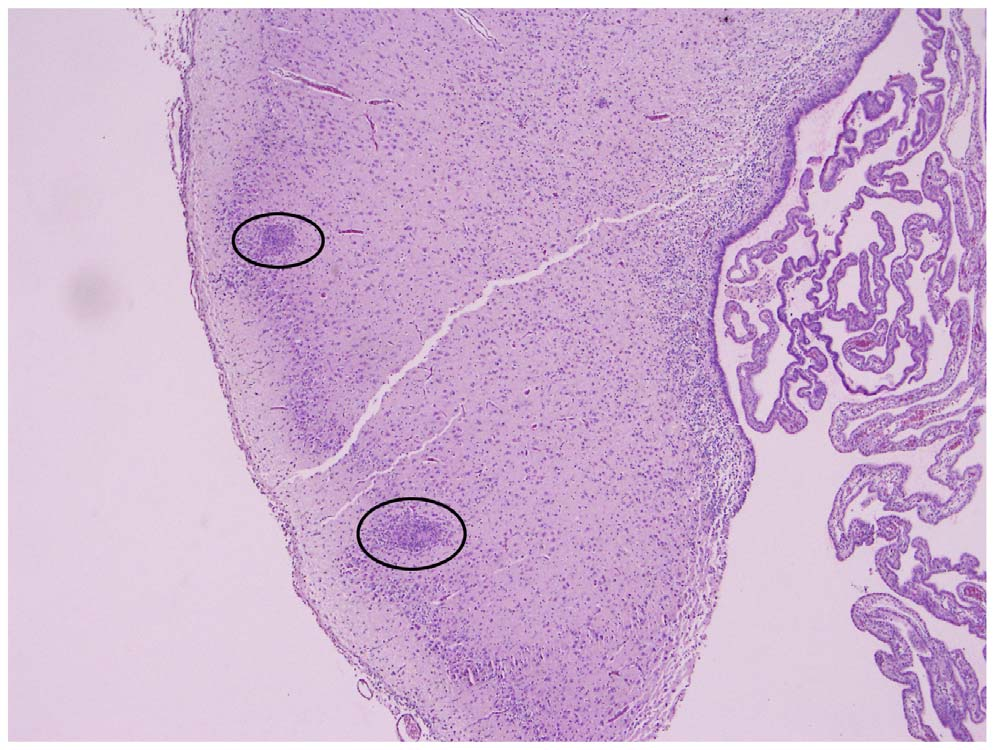
Mononuclear inflammatory infiltrates (circles). Cerebrum (4×10) of foetus 78649.1 (BTV-1 group, VI/PCR positive).

**Figure 7:**
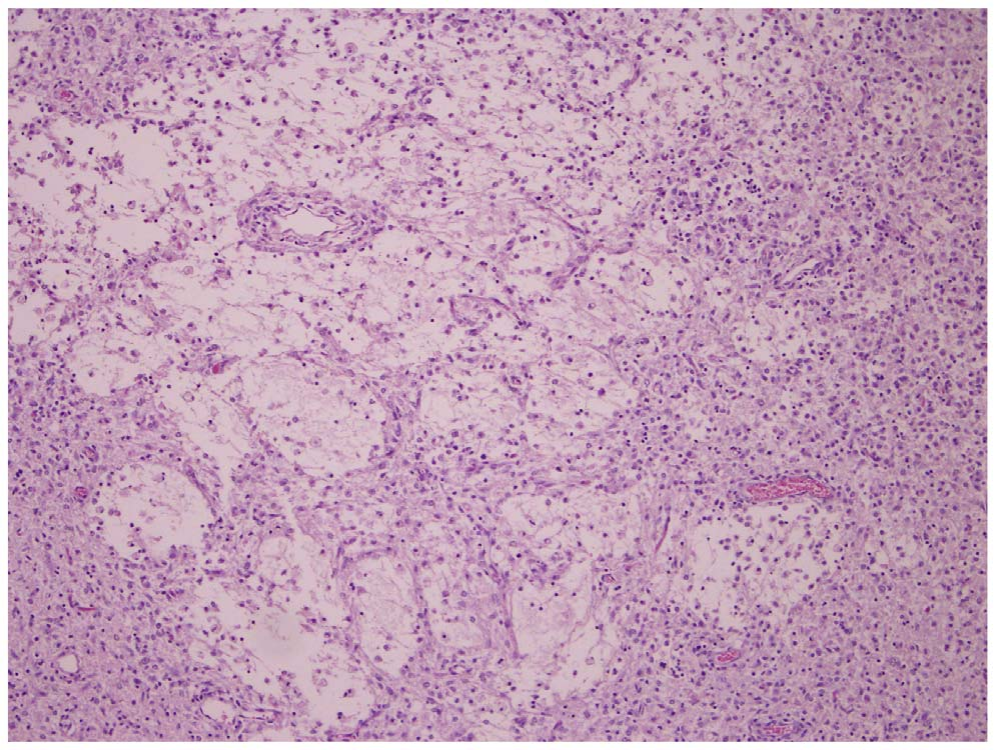
Necrosis and liquefaction of cerebral tissue. Cerebrum (10×10) of foetus 78655.1 (BTV-1 group, VI/PCR positive).

**Figure 8:**
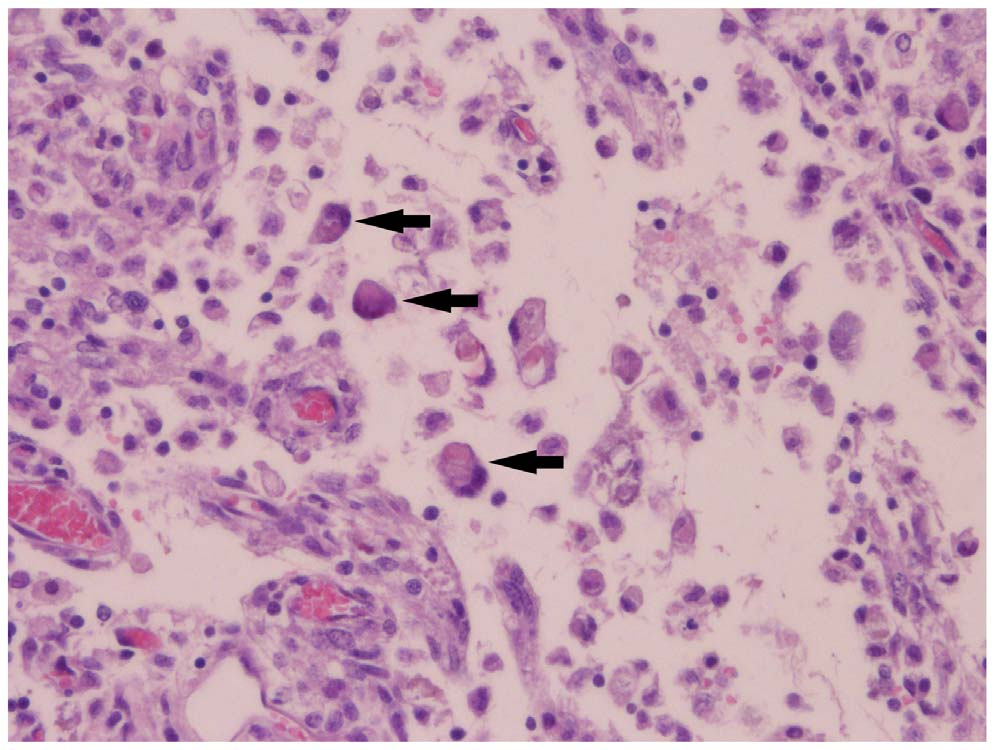
Necrosis, presence of multiple multinucleated giant cells (arrows), the lower one with vacuoles. Cerebrum (40×10) of foetus 78655.1 (BTV-1 group, VI/PCR positive).

**Figure 9:**
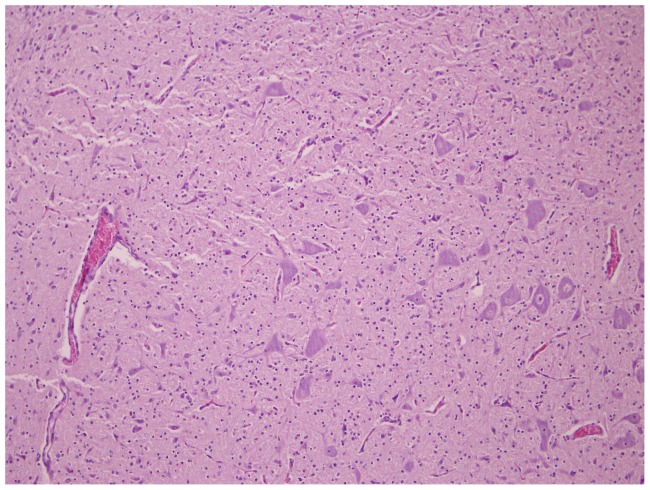
Normally developed cerebral tissue. Cerebrum (10×10) of foetus 78626.3(control of BTV-1 group, VI/PCR negative).

**Figure 10:**
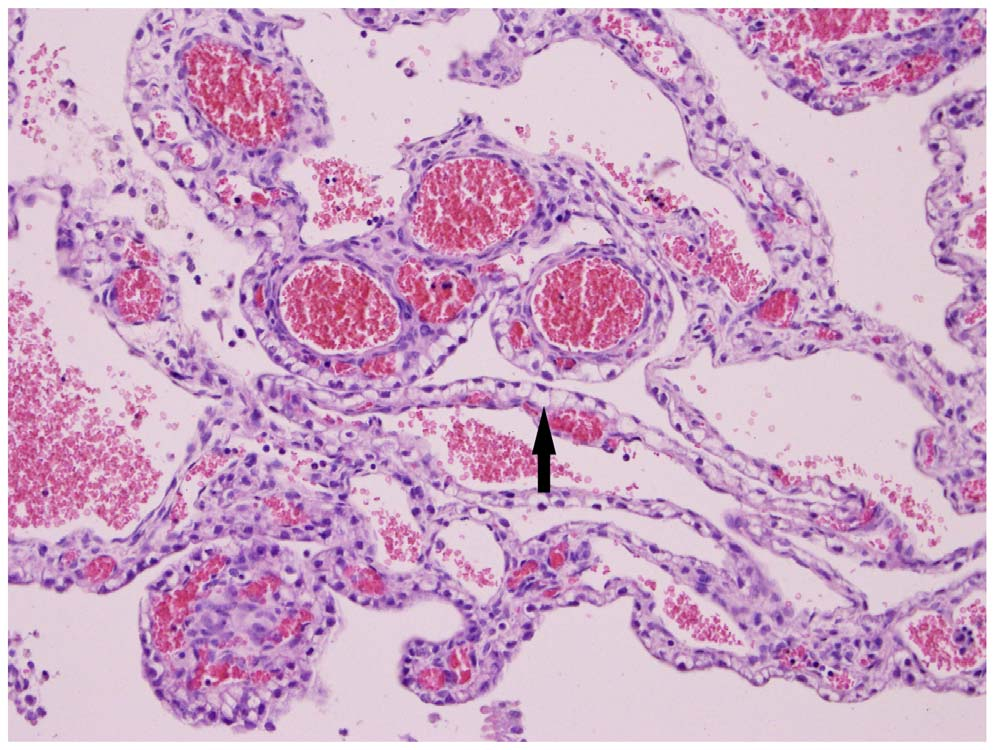
Ballooning degeneration of epithelium (arrow) and congestion of blood vessels. Plexus choroideus (20×10) of foetus 78631.3 (BTV-1 group, VI/PCR positive).

In the BTV-1 group, the incidence of microscopic findings was higher compared to the BTV-8 group (Figure 2); the lesions in the BTV-1 infected foetuses were more severe. Comparable to the BTV-8 group, lympho-histiocytic infiltrates were found in the meninges, but the presence of fibrinous exudate and giant cells (Figure 5) was unique to the BTV-1 group. In the parenchyma of the cerebrum and cerebellum, infiltrations of lymphocytes (Figure 6) and gliosis were found in respectively 13 and 10 of the 14 foetuses. In addition, large areas of liquefactive necrosis were seen in five foetuses, associated with giant cells (Figures 7–8), whereas this was not seen in the BTV-8 infected foetuses and in the non-infected foetuses (Figure 9). In the plexus choroideus of foetus 78631.3 (with hydranencephaly, ballooning degeneration of the epithelium and congestion of blood vessels were observed (Figure 10). The incidence of lymphoid depletion and reticular hyperplasia in the spleen was higher in the BTV-1 group.

## Discussion

The infection of 9 mid-term pregnant ewes with BTV-8 led to the transplacental infection of 12 foetuses (43%); 5/9 ewes (56%) transmitted the virus to their foetuses.

Yet, 6/9 (67%) mid-term pregnant ewes infected with BTV-1 transmitted the virus to 14 of their foetuses (82%). These results clearly demonstrate that, under the conditions in our experiment, BTV-1 is able to induce transplacental infection.

Furthermore, the central nervous system lesions observed in the BTV-1 infected foetuses were more severe than those seen in the BTV-8 infected foetuses. Hydranencephaly or porencephaly was observed in 8 of the BTV-1 infected foetuses, whereas in the BTV-8 group, only slight vacuolation of the parenchyma was observed in 3 foetuses.

Previous studies have demonstrated that BTV has a high affinity for undifferentiated cells in the brain. BTV infection of the ovine foetus around 50–60 days of gestation, a stage at which the neuronal and glial precursor cells have not yet differentiated and migrated to the cerebral cortex, led to destruction of the undifferentiated neuronal precursor cells resulting in massive necrosis and subsequent hydranencephaly [22], [29], [30]. Infection around 75 days of gestation led to milder and more localized lesions [29], [31].The findings reported here demonstrate that BTV-1 infection can induce severe necrotizing encephalitis even in foetuses which are infected at 70–75 days of gestation.

Nonetheless, the findings in our experiment do not match the experiences from the field: field investigations in 17 farms in Spain (including 88 dams and 93 calves) could not demonstrate any case of transplacental transmission in cattle upon natural infection with BTV-1 [32]. Several factors might be suggested to explain the difference between the findings in our experiment and in the field.

First of all, transplacental transmission of BTV is up to now mainly associated with modified live vaccines, which contain virus strains that have been attenuated through multiple -sometimes over 50- *in vitro* passages. Bluetongue viruses, like other RNA viruses, exist as a swarm of closely related sequence variants with one or several dominant master sequences (quasispecies) [33]. Changes in the environment can lead to selection of sequence variants with an improved fitness for the new environment. Hence, passages in an artificial culture system -like ECE or cell culture- may put the viruses through a genetic bottleneck, resulting in selection of minor viral variants which favour growth *in vitro*. These minor viral variants may also display phenotypical changes like changes in virulence, tissue tropism and ability to cross the placenta [34]. The viruses used in our experiment have been derived from field strains. However, our strains have been isolated in ECE and subsequently passaged once in Vero cells and once in BHK cells to obtain a challenge virus stock. Although the total number of passages was limited (three), and although for each passage a different culture system was used, the *in-vitro* passages might have resulted in a selection for minor viral variants that facilitate transplacental transmission.

Contradictory to this speculation are the findings by Flanagan et al. [21] and Roeder et al [35]. In their experiments with BTV-20 and BTV-11, a limited number of *in vitro* passages did not lead to selection for minor viral variants which can cross the ruminant.

A second explanation might be the lack of sufficiently targeted field data generated from larger surveys. Under natural circumstances, the transmission rate of BTV-1 might be low, and cases might be missed if there is no specific surveillance focussed on abortions or congenital malformations. The study in Spain, in which transplacental transmission could not be demonstrated, focussed on a limited number of cattle [32]; sheep were not investigated. In Italy, evidence of transplacental transmission of the BTV-2 and BTV-9 vaccine strains was only found after an extensive retrospective study in sheep, cattle, goats and buffalo [24].

A third explanation for the difference in findings might be the design of our experiment. The virus dose (4.0 log_10_ TCID_50_) and injection volume (10 ml) chosen for our experiment differ substantially from the dose and volume delivered by the bite of a *Culicoides* midge. The design of our experiment was chosen not to closely mimic natural infections; it was based on previous experiments that were successful in inducing transplacental transmission in a limited number of animals [26], [36], [37].

Infectious BTV-1 was detected in the blood and spleen samples of control ewe 78626. The timing of the first detection of BTV RNA (at 21 dpi) differed significantly from the BTV-1 inoculated ewes, which proves it unlikely that the ewe had accidentally been injected with BTV-1. The experiment was conducted during the vector free period in the winter of 2012, in insect secure isolation facilities. The only possibility for an insect to enter the isolation rooms was at the time of introduction of the ewes. However, the chance that a midge would enter the facilities in mid-winter, survive the cyfluthrin treatment and transmit BTV-1 to the controls is effectively zero. Therefore, the control ewe most likely was infected via direct contact. Vector-free horizontal transmission has been described previously for BTV-8 [26], [38], [39], and more recently for BTV-26 [40].

According to the Scientific Opinion of the Panel on Animal Health and Welfare of the European Food Safety Authority, there is no evidence that transplacental transmission of other serotypes than 8 occurs in regions where no modified live vaccines have been used. Based on this opinion, precautionary measures to prevent the spread of BTV by pregnant animals are restricted to BTV-8 [41]. In our experiments, we have irrevocably shown that under certain circumstances, BTV-1 can cross the placenta and infect the foetus, with severe pathological consequences. The viruses used in our experiment were cultured *in vitro*; extrapolation to the natural situation should therefore only be done with great caution. Yet, Bonneau et al (2010) [33] demonstrated that *Culicoides* feeding on low viraemic sheep can also result in fixing of a new genotype by founder effect. Therefore, more research comparing unpassaged and *in vitro* grown material is needed to better understand the drivers behind transplacental transmission of BTV.

## Conclusion

Both BTV-8 and BTV-1 were able to cross the placenta in 70–75 days pregnant ewes and infect the foetus. The incidence of transplacental transmission of BTV-1 was higher than that of BTV-8. BTV-1 infection induced a severe encephalopathy in 8/14 infected foetuses, whereas in the BTV-8 infected foetuses, only mild cerebral vacuolation was observed in 3/12 foetuses.
